# Beyond Accuracy: Clinical Outcomes of Computer Assisted Implant Surgery

**DOI:** 10.1002/cre2.70129

**Published:** 2025-05-16

**Authors:** Sofya Sadilina, Kay Vietor, Romain Doliveux, Adam Siu, Zhuofan Chen, Bilal Al‐Nawas, Nikos Mattheos, Allesandro Pozzi

**Affiliations:** ^1^ Clinic of Reconstructive Dentistry, Center for Dental Medicine University of Zürich Zürich Switzerland; ^2^ Private Practice Langen Germany; ^3^ Private Practice Mulhouse France; ^4^ Private Practice Neuenburg Am Rhein Germany; ^5^ Private Practice, Dental Implant Surgery Centre Hong Kong SAR China; ^6^ Guanghua School of Stomatology, Hospital of Stomatology Sun Yat‐sen University China; ^7^ Guangdong Provincial Key Laboratory of Stomatology Guangzhou China; ^8^ Department of Oral and Maxillofacial Surgery University Medical Center Mainz Mainz Germany; ^9^ Oral and Maxillofacial Surgery and Digital Implant Surgery Research Unit, Faculty of Dentistry Chulalongkorn University Bangkok Thailand; ^10^ Department of Dental Medicine Karolinska Institute Stockholm Sweden; ^11^ Department of Clinical Science and Translational Medicine University of Rome Tor Vergata Rome Italy; ^12^ Department of Periodontics and Oral Medicine University of Michigan USA; ^13^ Department of Restorative Sciences Augusta University Augusta Georgia USA; ^14^ Department of Restorative Dentistry and Biomaterials Sciences Harvard School of Dental Medicine Boston Massachusetts USA

**Keywords:** clinical outcomes, computer‐assisted implant surgery, dental implants, navigation, robotic surgery

## Abstract

**Objectives:**

Computer Assisted Implant Surgery (CAIS) with different technologies and modalities is becoming increasingly utilized in clinical practice. The aim of this White Paper was to synthesize evidence, reported experience, and best practices with regard to clinically relevant outcomes of static, dynamic, and robotic CAIS.

**Materials and Methods:**

A review of the literature compiled existing evidence from clinical studies up to November 2024, which was later discussed and synthesized into clinically relevant questions with a panel of international experts.

**Results:**

There is overwhelming evidence for the superiority of static, dynamic, and robotic CAIS with regard to the accuracy of implant placement and some limited evidence of superior esthetic outcomes. At the same time, outcomes related to implant primary stability, survival rates, intra‐ and postoperative complications, marginal bone loss, and peri‐implant tissue health appear similar between guided and non‐guided implant surgery, while efficiency is poorly defined and studied. The importance of accuracy in the execution of a comprehensive, prosthetically driven treatment plan is not reflected in most studies, which focus mainly on the assessment of procedures rather than entire treatment workflows. Such inherent limitations of available research might conceal some of the potential of guided CAIS.

**Conclusions:**

Guided CAIS can achieve at least as good clinical outcomes as non‐guided implant surgery. Studies that can assess the benefits of CAIS as part of a treatment workflow, rather than isolated procedures, could improve our understanding of the potential of these technologies.

## 1 Introduction

The wide introduction of digital tools and services in implant dentistry has led to a paradigm shift centered on detailed pre‐intervention selection design of implants, prosthesis, and transmucosal components, streamlining workflow and minimal invasiveness (Joda et al. 2024; Ricciardi et al. 2019). The utilization of Computer‐aided Design Implant Planning Software (CAD‐IPS) empowers the integration of radiographic, volumetric, surgical, prosthetic, and laboratory‐related data in a unified virtual environment (Jorba‐Garcia, Pozzi, et al. 2025; Lanis et al. 2017; Sadilina et al. 2024). Thus, by creating a three‐dimensional representation of the patients' anatomy, a CAD‐IPS facilitates comprehensive treatment planning, starting with the optimal design of the prosthesis and respective components based on patients' specific anatomic conditions (Mattheos et al. 2021), then extending to the ideal implant position and plan for the most suitable soft tissue surgical handling (Pedrinaci et al. 2024). The ultimate aim remains to reduce the invasiveness and intraoperative stress of procedures, increase patients' overall satisfaction, and improve long‐term treatment clinical outcomes (Schneider et al. 2018).

It is therefore evident that the ability to place the implant in the planned position with accuracy is a prerequisite for success with comprehensive treatment planning. As a result, Computer Assisted Implant Surgery (CAIS) is increasingly perceived as a critical link in the digital workflow in implant dentistry (Pedrinaci et al. 2024). At the same time, the accuracy of implant placement can only be meaningful as part of an evidence‐based comprehensive treatment plan. Thus, increased accuracy should be ultimately assessed by the overall improvements in the clinical outcomes it empowers. A large number of clinical trials has now established the superiority of static (Gourdache et al. 2024; Pozzi et al. 2014; Putra et al. 2022), dynamic (Jorba‐García et al. 2021; Schnutenhaus et al. 2021; Vinnakota et al. 2023; Yu et al. 2023), and robotic (Pimkhaokham et al. 2024) CAIS with regard to the accuracy of implant placement. However, most studies are limited to assessing measures of implant deviation (Pimkhaokham et al. 2024). Clinically relevant outcomes such as intra‐ and post‐operative complications, healing parameters, outcomes related to tissue stability, and health and aesthetics are scarcely being reported and are often considered secondary outcomes (Pimkhaokham et al. 2022). As this technology matures, it is imperative to examine whether and how the increased accuracy leads to more efficient treatments, improved treatment results, and overall increased patient benefits (Smitkarn et al. 2019). Therefore, a combination of clinical, radiographic, aesthetic, and technical outcomes would be required to advance our understanding of the benefits of CAIS and to determine its role in the digital workflow in the future.

The aim of this White Paper was to synthesize currently available evidence, reported experience, and best practices with regard to clinically relevant outcomes of static, dynamic, and robotic CAIS other than measures of accuracy. In doing so, the paper aspires to offer support for clinical decision‐making, as well as identify future directions and upcoming developments with the potential to impact clinical practice and treatment outcomes.

## 2 Search Strategy and Selection of the Studies

A comprehensive electronic search was conducted in MEDLINE via PubMed up to October 15, 2024 (Table 1) and supplemented by a manual search in the relevant journals. Additionally, cross‐reference checking was conducted in the bibliographies of all included studies and relevant reviews on the topic. The search was restricted to publications in English and only clinical trials on static, dynamic, or robotic CAIS were considered for this review. Systematic reviews of clinical trials on the topic were also assessed for relevant publications and/or data.

**Table 1:** Search strategies.

Database	
Medline (via PUBMED)	(“Surgery, Computer‐Assisted”[Mesh] OR therapy, computer‐assisted [Mesh] OR “navigation system*” OR “dynamic computer aided” OR “dynamic computer guided” OR “dynamic computer assisted” OR “static computer aided” OR “static computer guided” OR “static computer assisted” OR “robotic computer aided” OR “robotic computer guided” OR “robotic computer assisted”) AND (“Dental Implantation” [Mesh] OR “Dental Implants”[Mesh] OR “dental implant*” OR “implant placement” OR implantology)
Search date	October 15, 2024

Abbreviation: MeSH, medical subject headings.

## 3 Clinically Relevant Outcomes of Computer Assisted Implant Surgery

**Figure 1:**
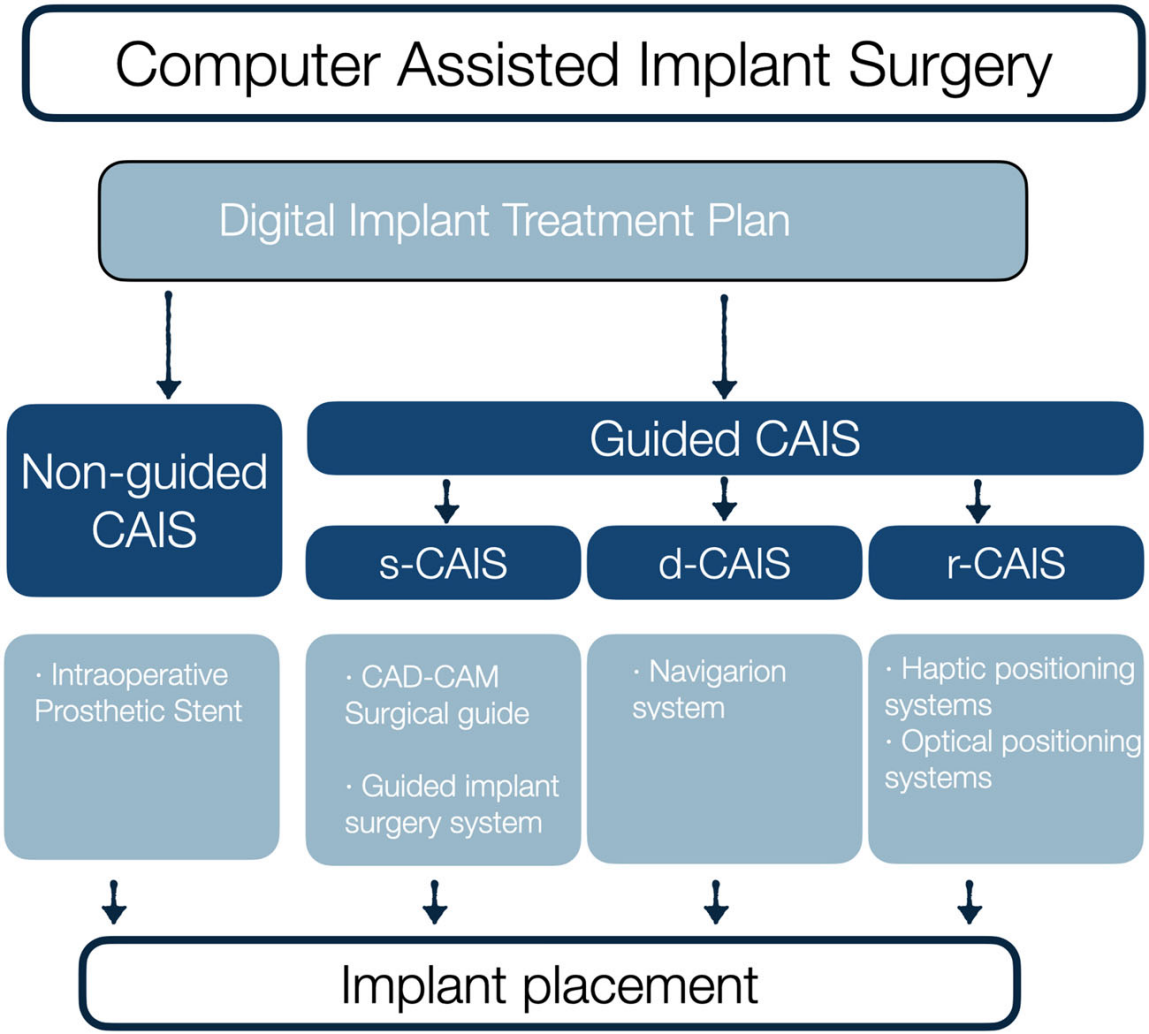
Overview of Computer Assisted Implant Surgery organization, adjusted from the Glossary of Computer‐assisted Implant Surgery and related terms. First edition (Jorba‐Garcia et al. 2025). CAD‐CAM, computer‐aided design–computer‐aided manufacturing; CAIS, computer assisted implant surgery; d‐CAIS, dynamic computer assisted implant surgery; r‐CAIS, robotic computer assisted implant surgery; s‐CAIS, static computer assisted implant surgery.

**Figure 2:**
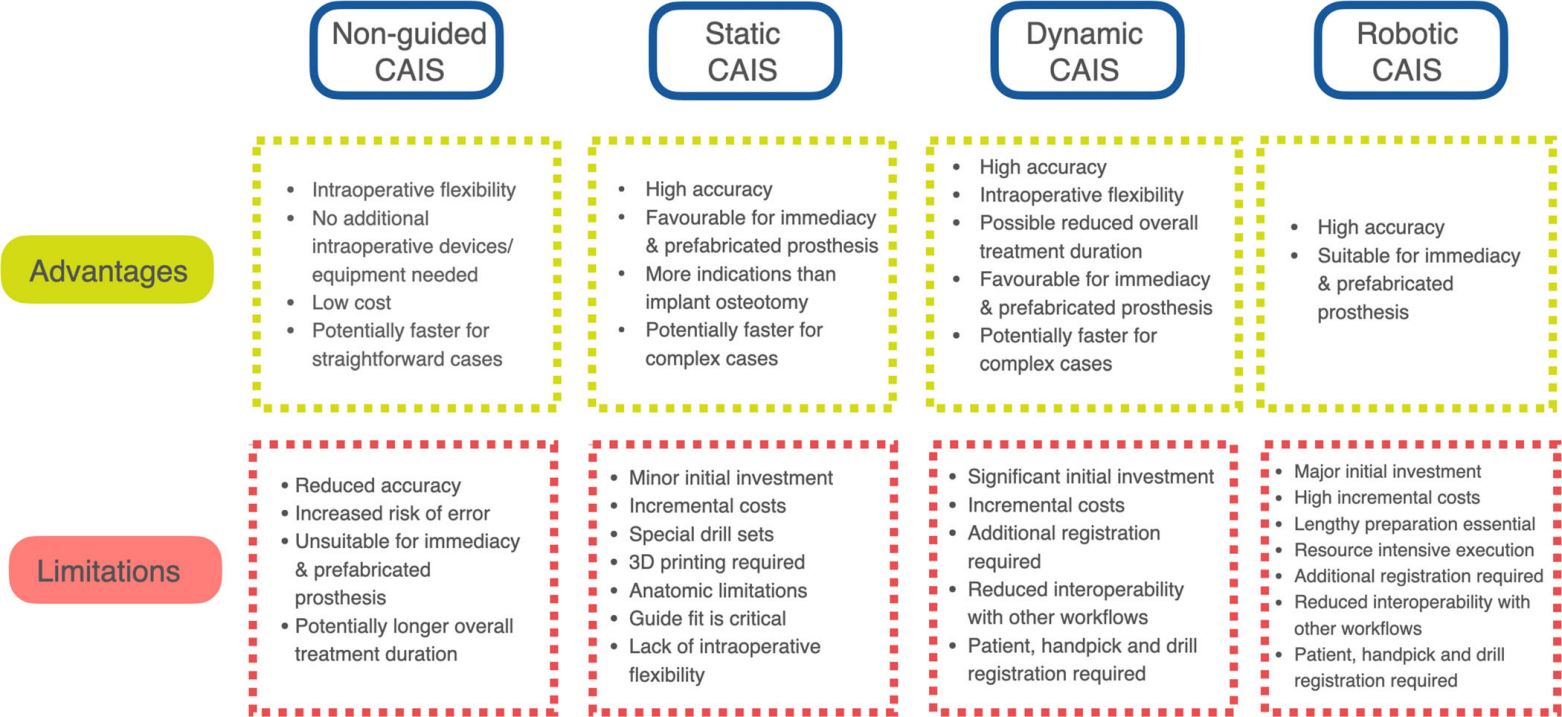
Overview of computer‐assisted implant surgery main workflows with reported advantages and limitations. CAIS, computer assisted implant surgery.

The manuscript followed the definition and organization of CAIS, as presented in the Glossary of Computer‐assisted Implant Surgery and related terms. First edition (Jorba‐Garcia et al. 2025) (Figure 1). Different sets of clinical and radiographic outcomes have been utilized to assess the success of implant treatment when CAIS workflows were utilized, which have led to the formulation of the focus questions of the current white paper (Table 2). The findings of this literature review were discussed with a group of expert clinicians with extensive clinical, teaching, and research experience in the application of CAIS and further synthesized with current best practices and expert opinion (Figure 2).

**Table 2:** Synthesis of the main clinically relevant outcomes of guided CAIS, as benchmarked against outcomes of non‐guided CAIS. Yellow marks outcomes in general “as good as”, green marks outcomes in general superior, and red inferior to those achieved with ng‐CAIS.

Outcomes	s‐CAIS	d‐CAIS	r‐CAIS
Accuracy	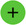	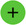	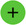
Complications	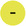	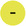	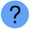
Esthetic outcomes	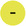	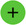	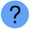
Implant stability	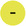	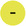	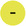
Implant survival rate	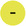	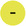	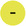
Marginal bone loss	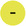	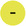	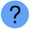
Peri‐implant soft tissue health	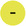	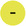	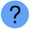
Time‐efficiency	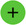	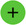	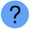

Abbreviations: CAIS, computer assisted implant surgery; d‐CAIS, dynamic computer assisted implant surgery; ng‐CAIS, non‐guided computer assisted implant surgery; r‐CAIS, robotic computer assisted implant surgery; s‐CAIS, static computer assisted implant surgery.

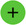
 ‐ Superior to ng‐CAIS

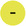
 ‐ As good as ng‐CAIS

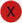
 ‐ Inferior to ng‐CAIS

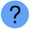
 ‐ No information

### 3.1 Is the Use of Guided CAIS Beneficial in Terms of Accuracy?

#### 3.1.1 Summary

Guided CAIS can achieve significantly higher accuracy compared to the free‐hand approach, as documented by a significant number of clinical trials, further supported by systematic reviews and meta‐analyses. At the same time, some meta‐analyses suggest marginal superiority of d‐CAIS (dynamic computer assisted implant surgery) to s‐CAIS (static computer assisted implant surgery), while emerging data suggest that r‐CAIS (robotic computer assisted implant surgery) could further increase the benchmarks of accuracy.

#### 3.1.2 Explainer

With the accumulation of a large volume of data from clinical trials, multiple systematic reviews and meta‐analyses have documented beyond doubt the higher accuracy achieved with both s‐CAIS (Pozzi et al. 2016; Putra et al. 2022; Romandini et al. 2023; Tahmaseb et al. 2018) and d‐CAIS (Pellegrino et al. 2021) when compared to a non‐guided approach. When comparing different guided CAIS approaches, some meta‐analyses have suggested an advantage of d‐CAIS over s‐CAIS (Li et al. 2024; Yu et al. 2023). However, a network meta‐analysis suggested that the combination of s‐ and d‐CAIS achieved significantly higher accuracy than either approach alone (Mahardawi et al. 2025). Finally, meta‐analyzed emerging evidence indicates r‐CAIS achieves significantly better outcomes in terms of accuracy than both s‐ and d‐CAIS (Khan et al. 2024; Khaohoen et al. 2024).

#### 3.1.3 Critique

Several randomized clinical trials (RCT) have documented the superiority of guided CAIS in terms of accuracy (Aydemir and Arısan 2020; Jorba‐García et al. 2023; Yotpibulwong et al. 2023), without however documenting any differences between the static or dynamic approach. Meta‐analyses, however, utilizing much larger samples suggest some advantage for d‐CAIS, even if marginal. With regard to r‐CAIS, however, significantly higher accuracy is being reported at clinical trials when compared to s‐CAIS (Jia et al. 2023) or s‐ and d‐CAIS (Shi et al. 2024), which suggests a larger magnitude of difference. Nevertheless, accuracy is only meaningful when related to a comprehensive treatment plan and the respective patient‐optimized implant position. Although the deviation in implant placement is being constantly reduced by CAIS technologies, the essential level of accuracy to fulfill the requirements of the treatment plan is unknown and varies in different clinical scenario. It is reasonable to anticipate that accuracy will play a more critical role in clinical scenario of higher complexity, for exampleesis In Fresh‐Fr, immediate loading with prefabricated prosthesis.

### 3.2 Does the Use of Guided CAIS Result in Higher Treatment Time‐Efficiency?

#### 3.2.1 Summary

The current evidence does not provide a definitive answer on whether guided CAIS improves the time‐efficiency of treatment. Guided CAIS is anticipated to be more time‐efficient compared to the free‐hand approach, but the benefit might mostly derive from empowering more efficient and less invasive workflows, rather than by shortening the surgical interventions.

#### 3.2.2 Explainer

The great majority of comparative studies do not suggest any clear advantage of guided CAIS over non‐guided approaches with regard to the duration of the surgeries, at least in partially edentulous patients (Almahrous et al. 2020; Kaewsiri et al. 2019; Pozzi et al. 2014; Sancho‐Puchades et al. 2019; Younes et al. 2019). The same appears to be largely the case when different guided CAIS approaches are compared with each other (Mangano et al. 2018; Mouhyi et al. 2019; Zhu et al. 2024). At the same time, surgical duration might be influenced by different parameters in different CAIS modalities such as time required for patient, handpiece and drill registration or connection errors, and potential adjustments for deficient guide fit in s‐CAIS. Nevertheless, comparisons limited to the surgical intervention might not adequately reflect the strength of guided CAIS in empowering more efficient and less invasive workflows. For example, when guided CAIS is combined with flapless placement, it can significantly shorten the duration of surgery as compared to the flapped non‐guided approach with a mean difference of 24 min, as shown in a recent meta‐analysis (Romandini et al. 2023). Comparative data in surgical duration including r‐CAIS are very scarce, but emerging evidence through a recent RCT indicates the robotic surgeries to be significantly longer than s‐ and d‐CAIS at least when single gaps are concerned (Shi et al. 2024).

#### 3.2.3 Critique

Time efficiency is one of the crucial factors in selecting a treatment approach. At the same time, efficiency implies the ability to achieve the treatment objectives with as minimal time, resources, energy, effort, and so forth, as possible. Thus, efficiency is best approached as a characteristic of the entire treatment workflow. Considering its relevance, efficiency in clinical settings remains poorly defined and inconsistently addressed in the literature. Defining efficiency or even “time‐efficiency” in a patient‐centered manner or at the workflow level is one of the most important challenges for research in CAIS in the future. Most comparative studies primarily assess the duration of surgery across different modalities. However, to qualify for such comparisons, the cases included should be comparable in terms of extent and complexity, with the majority of studies assessing single gaps or partially edentulous patients. Even so, the definition of the surgical duration among studies might differ, in particular with regard to the time spent for system‐specific adjustments or corrections. Some experts have argued that a meaningful approach to time efficiency should be conducted at the treatment level, possibly including wider parameters necessitated by the use of CAIS such as time and resources allocated to digital treatment planning and preparation or potential benefits of the use of CAIS in overall treatment duration, number of sessions or overall chairside time, as well as reducing invasiveness. Operators' “learning curves” and investment required to reach mastery with guided CAIS might be also a parameter to add to the time‐efficiency equation. s‐CAIS has been widely utilized for a long period of time, which has allowed the respective protocols and devices to reach a higher degree of efficiency than newer approaches. Nevertheless, technologies of d‐ and r‐CAIS are advancing fast with novel Artificial Intelligence‐driven registration protocols, which may reduce technical errors and optimize surgeons' experience and workflow. Carefully designed research assessment at the workflow level rather than studies comparing only surgical interventions might help better understand the conditions and clinical scenario where guided CAIS can increase time efficiency and maximize benefits to the clinician and the patient.

### 3.3 Does the Use of Guided CAIS Result in Fewer Intra‐Operative and Postoperative Complications?

#### 3.3.1 Summary

Although guided CAIS is reasonably anticipated to reduce the frequency of intra‐ and postoperative complications through achieving increased accuracy, this is not reflected in either comparative or non‐comparative studies. Current studies report the prevalence of such complications with the use of CAIS to be very low but no different from the benchmarks achieved with non‐guided implant surgery. At the same time, expert critique has pointed out the inherent limitations of comparative studies, which focus on the level of the surgical intervention, while most potential advantages of guided CAIS protocols lie in facilitating outcomes of advanced workflows such as immediacy and minimal invasiveness.

#### 3.3.2 Explainer

Systematic reviews of comparative studies have reported no significant differences in the frequency of intra‐ or postoperative complications when different CAIS approaches were compared with freehand surgery (Pimkhaokham et al. 2022). At the same time, the frequency of complications when reported in non‐comparative studies assessing CAIS protocols appears to be low and not in any way different than the benchmarks set for conventional implant surgery (Bolding and Reebye 2022; Ding et al. 2023; Jia et al. 2023; Pozzi et al. 2022).

Typical intraoperative complications reported include damage of sensitive anatomic structures, excessive hemorrhage, absence of implant stability (D'haese et al. 2012; Di Giacomo et al. 2012; Ko et al. 2021; Mouhyi et al. 2019), and bone dehiscence after implant placement. However, each CAIS approach might introduce its own specific set of intraoperative complications on top of what is conventionally encountered in implant surgery (Kaewsiri et al. 2019). Studies utilizing s‐CAIS have additionally reported in‐stability/misfit (Mangano et al. 2018; Mouhyi et al. 2019; Penarrocha et al. 2012) or fracture (Nocini et al. 2013) of the surgical guide and anatomic limitation/problematic access for s‐CAIS drills (Derksen et al. 2019; Lin et al. 2020). When dynamic CAIS was utilized, reported intraoral complications included in‐stability/misfit of the intraoral stent (patient tracker) (Aydemir and Arısan 2020) and loss of connection/tracking with different frequencies. Interestingly, the occurrence and/or consequences of registration errors are not reported, with the exception of one case encountered with r‐CAIS in a recent RCT where the deviation of the drill on the robotic arm remained high after registration, thus a freehand approach was used (Shi et al. 2024).

Likewise, clinical studies have not shown any difference in terms of adverse events in the immediate post‐surgical healing period, survival, and osseointegration of implants placed with CAIS or freehand protocols, while complications reported in non‐comparative studies with CAIS such as failure of osseointegration (Pozzi et al. 2021) appear within the anticipated range defined by conventional implant surgery.

#### 3.3.3 Critique

As guided CAIS can deliver significantly higher accuracy than freehand placement, it would be reasonable to anticipate fewer intra‐operative complications, at least with regard to the frequency of damaging sensitive anatomic structures for example. The fact that this is not reflected in the currently available studies could be attributed to several factors, including limitations of common research designs. First, the currently established safety standards for the use of CAIS dictate that implants be planned with at least a 2 mm distance from any sensitive anatomic structures (Gallucci et al. 2018; Tahmaseb et al. 2018), similar to conventional implant surgery. Applying such a safety margin has contributed to a very low frequency of intraoperative complications for both guided and non‐guided CAIS protocols, with any differences in outcomes—if they exist—most likely being too small to be statistically significant within the realistically achievable sample sizes in comparative studies. At the same time, collective analysis of large data sets from different studies might be not meaningful, given the large diversity of techniques, devices, and protocols utilized under CAIS. Furthermore, most studies have been conducted in university hospital centers by clinicians experienced in both CAIS and non‐guided implant surgery. Although the use of CAIS should not be perceived as a remedy for a lack of experience, the high skill level of clinicians performing surgeries in most clinical trials indicates careful risk assessment prior to all surgeries, regardless of the protocol, resulting in minimal intraoperative complications. At the same time, expert critique has highlighted some inherent limitations of comparative research, in particular RCTs, which can only assess procedures with similar anatomic and surgical indications. There is little rationale as to why any postsurgical healing period would differ when CAIS is used if the procedure in both groups includes elevating a flap in a comparable location and surgical extent, preparation of the osteotomy with drills, implant placement, and consecutive suturing. Since different CAIS approaches have distinct indications and contraindications that impact the patient selection, a randomized trial would only be possible for patients who fulfill “a minimum common denominator” of eligibility across all methods. This would exclude clinical scenario where due to specific indications one approach would be favored against the other. However, significant advantages and potential risks of CAIS lie at the level of the workflow rather than the intervention itself, such as facilitating flapless surgery (Romandini et al. 2023), a factor that comparative studies may not adequately capture.

### 3.4 Does the Use of Guided CAIS Lead to Superior Esthetic Outcomes?

#### 3.4.1 Summary

While guided CAIS is reasonably expected to increase esthetic outcomes empowering higher implant position accuracy as part of digital smile design, for example, there is little scientific documentation. In a few medium‐term observations, guided CAIS demonstrated statistically significantly higher Pink Esthetic Scores (PES) compared to the non‐guided approach.

#### 3.4.2 Explainer

Several comparative studies have evaluated aesthetic outcomes of guided CAIS and the free‐hand approach. One RCT showed higher PES for single implants in the esthetic zone placed with s‐CAIS as opposed to free‐hand (Hanozin et al. 2022), while another comparative study concluded likewise that implants placed with either d‐ or s‐CAIS demonstrated statistically significantly higher PES scores to non‐guided CAIS after 4 years of observation (Heng et al. 2024). Furthermore, one non‐comparative study with implants in the anterior maxilla documented higher PES in implants with less positional deviation (Fürhauser et al. 2015). Meanwhile, prospective case series with d‐CAIS have documented increasing PES from placement to restauration and 1‐year postoperatively, from 8.22 ± 1.19 to 9.92 ± 1.16 and 12.34 ± 1.41, respectively (Pozzi et al. 2021), and from 9.51 ± 0.69 at implant placement to 12.84 ± 0.91 at the 1‐year follow‐up (Pozzi et al. 2022).

#### 3.4.3 Critique

Esthetic outcomes are increasingly important aspects of treatment success, as patient's esthetic expectations continue to rise. A fully digital workflow facilitates communication and expectation management between patients and dentists through Digital Smile Design. At the same time, guided CAIS plays a crucial role in ensuring accurate implant placement in the patient‐optimized 3D position, which is essential for achieving high aesthetic outcomes. It is important to note that esthetic outcomes are multifactorial, measured by “white” (e.g., prosthesis design, shape, color, tone) and “pink” (i.e., tissue consistency, morphology, color) aspects, as well as patients' perception and overall satisfaction. Whether guided CAIS can help achieve higher esthetic outcomes largely depends on the overall comprehensive plan and the entire workflow followed, of which CAIS is only one of several essential steps.

### 3.5 Does the Use of Guided CAIS Lead to Better Implant Stability?

#### 3.5.1 Summary

The stability of implants placed with guided CAIS is moderately studied. Although few studies might favor one or the other modality, most studies have documented that implants placed with guided CAIS achieve similar clinical stability to those placed with non‐guided protocols. It remains unclear if there is a difference between the guided CAIS approaches.

#### 3.5.2 Explainer

Some comparative studies assessing different outcomes related to implant stability achieved with non‐guided with s‐CAIS (Hanozin et al. 2022) and d‐CAIS (Wei et al. 2022) have shown no difference, while another comparative study favored THE stability of non‐guided placement over s‐CAIS (Smitkarn et al. 2019). At the same time, when d‐ and s‐CAIS were compared, one study found significantly higher primary stability of implants placed with s‐CAIS (Liu et al. 2024). It should be noted however that all implants in the above studies have achieved high levels of primary stability, as measured by insertion torque or implant stability quotient (ISQ). One non‐comparative study with d‐CAIS reported a mean insertion torque value of 60.7 ± 6.2 Ncm in post‐extraction sockets and 62.4 ± 8.2 Ncm in healed sites (Pozzi et al. 2021), while another prospective study with d‐CAIS and immediately placed implants reported a mean ISQ of 60.74 (Wei et al. 2022). Other studies with d‐CAIS have reported a mean insertion torque value of 49.0 ± 5.32 Ncm and a mean ISQ of 73.0 ± 5.71 (Pozzi et al. 2022) and 71.1 ± 2.8 (Pozzi et al. 2021).

#### 3.5.3 Critique

Primary implant stability is an important clinical parameter, particularly in cases of immediate implant placement and loading with temporary or permanent restoration. Primary stability depends on several factors from implant characteristics, surgical technique, and osteotomy protocol to bone quantity and quality. The digital treatment plan allows for the assessment of many of these factors. In particular, pre‐surgical assessment of bone volume and density can direct the surgeon to the selection of implant design and dimensions, proper osteotomy protocol, and implant position and thus ensure essential conditions for primary stability before commencing the surgery. It is unclear whether the use of guided CAIS alone can influence primary stability, but the overwhelming majority of studies show that implants placed with guided CAIS consistently achieve high levels of stability, at least as much as would be anticipated with non‐guided placement. The fact that d‐CAIS allows the surgeon's full tactile perception while conducting the osteotomy might allow minor intraoperative modifications, which could increase primary stability, such as changes in the osteotomy protocol. At the same time, the limited tactile perception while using s‐CAIS might conceal conditions of soft bone or reduce primary stability from the surgeon unless the surgical guide is removed. It is reasonable to anticipate that guided CAIS would have a more important role toward securing primary stability in complex cases, where stability can be reached only in specific positions with a narrow margin for error, such as in extraction sockets or compromised bone, especially if the plan involves immediate loading.

### 3.6 Does the Use of Guided CAIS Increase the Implant Survival Rate?

#### 3.6.1 Summary

Implants placed with guided CAIS are documented to achieve high survival rates, comparable to those achieved with non‐guided placement. Neither specific benefit nor detriment to the survival of the implants is anticipated using guided CAIS on the basis of the available data.

#### 3.6.2 Explainer

Comparative studies between implants placed with guided and non‐guided CAIS have shown equally high survival rates (Bernard et al. 2019; Cristache et al. 2021; Kunavisarut et al. 2022; Penarrocha et al. 2012; Smitkarn et al. 2019), although most prospective studies are limited to observations between 3 and 12 months, with one extending to 24 (Cristache et al. 2021) and one to 36 months (Bernard et al. 2019). A recent systematic review found a mean survival rate of 97% for implants placed with guided CAIS, based on a meta‐analysis of 16 studies. The review concluded that survival rates with guided CAIS are no different from the currently established benchmarks for non‐guided implant survival (Aghaloo et al. 2023).

#### 3.6.3 Critique

Implant survival is influenced by many factors, from pre‐surgical assessment and planning to surgical and restorative protocols and maintenance. Implants placed with guided CAIS show survival rates similar to those placed freehand at least in the short‐ to medium‐term observation periods. Implant survival in the long term (5–10 years) might be influenced by other factors than the CAIS protocol followed. There is no reason to anticipate either benefit or detriment to long‐term survival rates of implants placed with the same standards of patient selection and treatment planning, regardless of the guided CAIS modality utilized.

### 3.7 Does the Use of Guided CAIS Result in Lower Marginal Bone Loss?

#### 3.7.1 Summary

There is limited data suggesting the potential of guided CAIS to reduce marginal bone loss (MBL) in implants placed in the esthetic zone. In general, few studies that have reported marginal bone loss in the short term appear well within the benchmarks reached with non‐guided CAIS.

#### 3.7.2 Explainer

One recent comparative cross‐sectional study showed a significantly less marginal bone loss for implants placed with guided CAIS in the esthetic zone (guided CAIS: −0.29 ± 0.56 mm/non‐guided CAIS: −0.74 ± 0.71 mm) after an average of 4 years post‐loading (Heng et al. 2024). Two non‐comparative studies have provided assessments of MBL with d‐CAIS at the 1 year post loading reporting an average of −0.63 ± 0.25 mm in the aesthetic zone (Pozzi et al. 2021) and an average of −0.72 ± 0.26 mm (Pozzi et al. 2022).

#### 3.7.3 Critique

The marginal bone level is an important parameter of the success of implant therapy and can be influenced by many factors in both the short and long term. Early MBL can result from physiological bone remodeling due to factors related to local anatomy, surgical intervention (Acharya et al. 2019), or the design of abutment (Souza et al. 2018) or prosthesis (Strauss et al. 2024) used during the healing period. Prosthesis design (Mattheos et al. 2021) can be linked to MBL in the medium term as well as linked to bone loss related to peri‐implantitis in the long term (Katafuchi et al. 2018). Although multifactorial, peri‐implant MBL is strongly associated with implant position and placement depth, as well as prosthesis design elements such as the emergence angle (Strauss et al. 2024) and the height of the supracrestal complex (Puisys et al. 2023). Thus, the marginal bone level can be drastically influenced by the design and execution of a comprehensive treatment plan. To the extent that guided CAIS can empower the accurate execution of a well‐planned implant prosthesis, it is reasonable to expect that it has the potential to reduce marginal bone loss and benefit all outcomes related to the long‐term health of peri‐implant tissues. This effect however might be difficult to document in reasonably sample‐sized comparative studies, where the same high standards of planning and design of the restoration have to be applied for all implants. A well‐planned comprehensive implant prosthesis supported by the properly positioned implant in a well‐maintained and motivated patient will have high long‐term success regardless of whether the implant being placed guided or not. Guided CAIS however would increase our ability to place the implant in the optimal position and thus seamlessly execute the treatment plan.

### 3.8 Is the Use of Guided CAIS Beneficial for Peri‐Implant Soft Tissue Health?

#### 3.8.1 Summary

Very few studies have assessed peri‐implant tissue health in implants placed with guided and non‐guided CAIS, without showing any significant difference.

#### 3.8.2 Explainer

One recent comparative cross‐sectional study found no significant difference in clinical parameters of peri‐implant tissue health (bleeding on probing, probing depths) for implants placed with guided CAIS in the esthetic zone after an average of 4 years post‐loading (Heng et al. 2024). The same study classified implants as healthy, with mucositis, or with peri‐implantitis based on the case definitions from the World Workshop on the Classification of Periodontal and Peri‐Implant Diseases and Conditions (Berglundh et al. 2018), without however showing any difference between guided and non‐guided CAIS implants (Heng et al. 2024). A non‐comparative study evaluated d‐CAIS in 18 patients with different clinical scenarios. Two cases of mucositis due to root‐shield exposure occurred (Pozzi et al. 2022).

Few non‐comparative studies have reported plaque accumulation and bleeding scores around implants placed with d‐CAIS 1 year after placement (Pozzi et al. 2022; Pozzi et al. 2021), which however would fall within the current benchmarks anticipated for implant therapy regardless of the CAIS modality followed.

#### 3.8.3 Critique

Peri‐implant tissue health is a primary criterion for the success of implant therapy and one of the most important parameters to assess and maintain. At the same time, inflammation of the peri‐implant tissue, albeit plaque‐induced, is a multifactorial condition influenced by many factors from patients' systemic and behavioral background to restorative design, oral hygiene, and maintenance protocols. Similar to marginal bone loss, peri‐implant tissue inflammation is strongly associated with the design of the prosthesis, either as mucositis (Rungtanakiat et al. 2023) or peri‐implantitis (Katafuchi et al. 2018). With malposition of the implant being a major reason for compromised prosthesis design (Puisys et al. 2023), guided CAIS could help maintain healthy peri‐implant tissues by empowering accurate placement of the implant in the patient‐optimized, three‐dimensional prosthetic‐driven position. Again, the use of guided CAIS would not be the key determinant here, but rather the presence of a comprehensive prosthetic‐driven treatment plan, with guided surgery empowering its seamless execution. Thus, when equally high standards of prosthetic‐driven treatment plan and restoration have been applied for implants placed with guided and non‐guided CAIS, reasonably sample‐sized comparative studies might not be anticipated to show any difference with regard to peri‐implant tissue health outcomes, in particular when single or straightforward implants are being assessed.

## 4 Discussion

As the increased accuracy of implant placement with guided CAIS is overwhelmingly documented, further assessing clinically relevant outcomes becomes the essential next step. The present White Paper aimed to summarize the current evidence with regard to some important clinically relevant outcomes and synthesized it with current best practices and experts' opinions while at the same time investigating the limitations of the available scientific research and study designs.

Reviewing the evidence with regard to the most important clinical outcomes, guided CAIS appears to be at least as good as non‐guided CAIS, with regard to implant survival, primary stability, intra‐ and postoperative complications, and peri‐implant tissue health. Some advantages have been reported for guided CAIS in the esthetic zone, where it can lead to better outcomes in terms of pink esthetic scores and marginal bone levels than the non‐guided approach. At the same time, the ability to assess the benefits of guided CAIS may be limited by inherent constraints of comparative studies, particularly randomized clinical trials, as the focus on comparing procedures within otherwise identical workflows restricts the indication‐driven application of the technology.

Every technological advancement implemented in implant therapy must lead to clear improvements in the care delivered and measurable benefits for the patient. Thus, it is only reasonable to anticipate that technologies leading to more accurate implant placement should also improve clinical outcomes. At the same time, it becomes apparent that accuracy in itself is meaningless outside the framework of a comprehensive, evidence‐based treatment plan and a prosthetic‐driven implant position. Guided implant placement will not compensate for a deficient treatment plan, on the opposite it might amplify its consequences. This might well illustrate one main reason for the lack of major differences with regard to clinical outcomes in comparative studies: In the presence of an evidence‐based restorative‐driven digital treatment plan and consequently an appropriate restoration, there is little reason to anticipate long‐term clinical outcomes to be different whether implants were placed with guided or non‐guided CAIS. Clinical trials comparing outcomes of guided and non‐guided CAIS would have to maintain the same strict standards with regard to both comprehensive digital plan as well as restoration design, thus significant differences in outcomes such as survival, primary stability, or long‐term peri‐implant tissue health are not anticipated, especially if the study involves single implants, as is the case for most comparative trials. The strength of guided CAIS is not that it compensates for deficient planning but rather that it empowers the accurate execution of the appropriate plan, and this might be more critical as the complexity of the procedure rises. Placing a single implant within the tolerance margins of a restorative‐driven treatment plan might be well manageable by a trained clinician with analogue aids. However, this may be much more challenging in complex clinical cases, such as multiple sites, flapless placement in fresh extraction sockets, complete arch restorations, or fully edentulous patients—precisely where guided CAIS offers major advantages. The use of d‐CAIS has been reported as a critical link in the workflow, enabling complete‐arch implant placement with immediate loading using a digitally prefabricated fixed dental prosthesis (Pozzi et al. 2024). It was the experts' view that non‐guided CAIS is increasingly becoming the minimum standard of care, ensuring that a comprehensive, prosthetically driven digital treatment plan precedes every implant intervention. Although non‐guided CAIS and implant placement might suffice to ensure appropriate clinical outcomes in straightforward cases, the importance of guided CAIS would increase together with the complexity of the treatment protocol. In the future, studies comparing clinical outcomes of entire workflows rather than surgical procedures might help better assess the benefits of guided CAIS.

## 5 Conclusions

The use of guided Computer‐assisted implant surgery is shown to deliver similar clinical outcomes in terms of implant survival, primary stability, intra‐ and postoperative complications, and peri‐implant tissue health with non‐guided CAIS. It has the potential to increase time efficiency of the surgical intervention in cases of increased complexity, while it might result to better clinical outcomes than non‐guided CAIS in the esthetic zone, in terms of marginal bone levels and pink esthetic score. The significantly higher accuracy of implant placement compared achieved by guided CAIS can be essential for the execution of an evidence‐based, prosthetic‐driven treatment plan, especially in cases of higher complexity, such as placement in extraction socket, flapless placement, immediate loading, and fully edentulous patients. Thus, a major benefit of guided CAIS lies in its ability to empower successful clinical outcomes of entire workflows, something that current comparative clinical studies have not yet attempted to assess. In the future, new patient‐centered research approaches focused on the entire treatment workflow rather than the specific intervention might provide deeper insights into the clinical benefits and indications of the use of guided CAIS.

## Author Contributions

Conceptualization: Bilal Al‐Nawas, Nikos Mattheos, and Alessandro Pozzi. Data curation: Sofya Sadilina. Formal analysis: Sofya Sadilina and Nikos Mattheos. Investigation: Sofya Sadilina. Methodology: Bilal Al‐Nawas, Nikos Mattheos, and Alessandro Pozzi. Project administration: Nikos Mattheos. Resources: Bilal Al‐Nawas. Supervision: Bilal Al‐Nawas and Nikos Mattheos. Validation: Kay Vietor, Romain Doliveux, Adam Siu, and Zhuofan Chen. Visualization: Sofya Sadilina and Nikos Mattheos. Writing – original draft: Sofya Sadilina and Nikos Mattheos. Writing – review and editing: Sofya Sadilina, Nikos Mattheos, Kay Vietor, Romain Doliveux, Adam Siu, Zhuofan Chen, Bilal Al‐Nawas, and Alessandro Pozzi.

## Conflicts of Interest

The authors declare no conflicts of interest.

## Data Availability

The data that support the findings of this study are available from the corresponding author upon reasonable request.
